# Genome-wide identification and characterization of *SnRK2* gene family in cotton (*Gossypium hirsutum* L.)

**DOI:** 10.1186/s12863-017-0517-3

**Published:** 2017-06-12

**Authors:** Zhao Liu, Xiaoyang Ge, Zuoren Yang, Chaojun Zhang, Ge Zhao, Eryong Chen, Ji Liu, Xueyan Zhang, Fuguang Li

**Affiliations:** State Key Laboratory of Cotton Biology, Institute of Cotton Research, Chinese Academy of Agricultural Sciences, Anyang, 455000 China

**Keywords:** SnRK2 kinase family, Upland cotton, Phylogenetic analysis, Expression analysis

## Abstract

**Background:**

Sucrose non-fermenting-1-related protein kinase 2 (SnRK2) is a plant-specific serine/threonine kinase family involved in the abscisic acid (ABA) signaling pathway and responds to osmotic stress. A genome-wide analysis of this protein family has been conducted previously in some plant species, but little is known about *SnRK2* genes in upland cotton (*Gossypium hirsutum* L.). The recent release of the *G. hirsutum* genome sequence provides an opportunity to identify and characterize the SnRK2 kinase family in upland cotton.

**Results:**

We identified 20 putative SnRK2 sequences in the *G. hirsutum* genome, designated as *GhSnRK2.1* to *GhSnRK2.20*. All of the sequences encoded hydrophilic proteins. Phylogenetic analysis showed that the *GhSnRK2* genes were classifiable into three groups. The chromosomal location and phylogenetic analysis of the cotton *SnRK2* genes indicated that segmental duplication likely contributed to the diversification and evolution of the genes. The gene structure and motif composition of the cotton *SnRK2* genes were analyzed. Nine exons were conserved in length among all members of the *GhSnRK2* family. Although the C-terminus was divergent, seven conserved motifs were present. All *GhSnRK2s* genes showed expression patterns under abiotic stress based on transcriptome data. The expression profiles of five selected genes were verified in various tissues by quantitative real-time RT-PCR (qRT-PCR). Transcript levels of some family members were up-regulated in response to drought, salinity or ABA treatments, consistent with potential roles in response to abiotic stress.

**Conclusions:**

This study is the first comprehensive analysis of *SnRK2* genes in upland cotton. Our results provide the fundamental information for the functional dissection of *GhSnRK2s* and vital availability for the improvement of plant stress tolerance using *GhSnRK2s*.

**Electronic supplementary material:**

The online version of this article (doi:10.1186/s12863-017-0517-3) contains supplementary material, which is available to authorized users.

## Background

Plants experience various biotic and abiotic stresses in their natural environment, such as drought and salt stress. Abscisic acid (ABA) plays an important role in plant response to drought and high salinity. ABA was first discovered in the 1960s and isolated from cotton as a promoter of leaf abscission [1, 2]. ABA also regulates diverse plant growth and developmental processes, including seed development, stomatal closure, promotion of seed dormancy and reproduction [3–6].

Protein phosphorylation/dephosphorylation is an important mechanism for mediating intracellular responses, i.e. responses to hormonal, pathogenic, and environmental stresses [7]. Various studies in plants have indicated that protein kinases are involved in stress signaling via phosphorylation/dephosphorylation, for example, the plant mitogen-activated protein kinase (MAPK) [8], calcium-dependent protein kinase (CDPK) [9], and sucrose non-fermenting1 (SNF1) kinase families. The SNF1 kinase family is known as SNF1 protein kinases in yeasts, AMP-activated protein kinases in mammals, and SNF1-related protein kinases (SnRKs) in plants [10]. All kinases in yeasts and mammals play key roles in carbohydrate metabolism, whereas plant SnRKs may function as interfaces between stress and metabolic signaling pathways [11–13].

The SnRK protein kinases are classified into three subfamilies: SnRK1, SnRK2 and SnRK3 [12, 14]. *SnRK1* is the plant homolog of the yeast *SNF1* gene and plays a role in global regulation of carbon and nitrogen metabolism [15]. The SnRK2 and SnRK3 subfamilies are unique in plants and may be involved in responses to environmental stress. Members of the SnRK2 subfamily contain a highly conserved kinase domain, an ATP-binding domain, and a serine/threonine active site [16]. SnRK3 kinases are known as calcineurin B-like calcium sensor-interacting protein kinases. The kinase domain of this subfamily contains a binding site for calcium-binding proteins and calcium-sensitive calcineurin B-like proteins in the C-terminal region, which combine to activate the protein kinase [17]. The best known member of this group is the Salt Overly Sensitive 2 kinase, which is required for sodium and potassium ion homeostasis and salt tolerance [18, 19]. The SnRK1 and SnRK2 subfamilies of protein kinases are similar in their catalytic domain but have divergent C-terminal domains, whereas the SnRK3 subfamily is structurally distinctive [20]. In the present study we focused on SnRK2 family members in *G. hirsutum*.

The first *SnRK2* cDNA clone (PKABA1) was isolated from an ABA-treated wheat embryo cDNA library, which was induced by dehydration, cold, salinity, and osmotic stresses [21, 22]. In a recent study, overexpression of *TaSnRK2.4* resulted in delayed seeding establishment, longer primary roots and enhanced tolerance to abiotic stresses in *Arabidopsis* [23]. Transgenic experiments indicated that *TaSnRK2.7* and *TaSnRK2.8* significantly increased tolerance to drought, salt and cold stress in *Arabidopsis* [24, 25]. In the past decade, SnRK2 kinases have been identified in diverse plants, such as *Arabidopsis*, rice, maize, soybean and tobacco [26]. There are ten *SnRK2* genes in *Arabidopsis*; among these, nine genes (except *SnRK2.9*) are activated by hyperosmotic and salinity stress, and five out of nine are also activated by ABA, whereas none of these genes are activated by cold stress [27]. Moreover, SnRK2.6/SRK2E//OST1 is involved in ABA regulation during stomatal closure and ABA-mediated gene expression [28, 29]. Overexpression of *SnRK2.8* enhances drought tolerance in *Arabidopsis* [30]. In rice, all ten *SnRK2* members have been isolated and found to be activated by hyperosmotic stress but only three (*SAPK8, SAPK9* and *SAPK10*) are activated by ABA [31]. Overexpression of *SAPK4* significantly enhances the salt tolerance of transgenic plants [32]. In maize, 10 *SnRK2* genes have been cloned and most of these are activated by one or more abiotic stresses [33]. *ZmSPK1*, a SnRK2 homolog in maize, is expressed in different plant tissues including roots, leaves and reproductive organs as well [34]. In soybean, four SnRK2 members have been isolated, where all these are induced by hyperosmotic stress [35, 36]. The expression level of only *SPK3* and *SPK4* increases in response to dehydration and high salinity [35]. A 42 kDa protein kinase (NtOSAK), belonging to the SnRK2 family, is activated by osmotic stress in tobacco [31]. Overexpression of *NtSnRK2.1* confers enhanced salt tolerance in tobacco, which suggests that NtSnRK2s are involved in abiotic stress response pathways [37].

Cotton is an economically important and a model polyploid crop [38, 39]. Upland cotton (*G. hirsutum*), an allotetrapoid species (A_t_A_t_D_t_D_t_, 2*n* = 4*×* = 52, where ʻt’ stands for tetraploid), is the widely cultivated cotton species that accounts 90% of total world cotton production [40–42]. Only one *SnRK2* gene has been identified from upland cotton [42]. Nevertheless, systematic analysis of SnRK2 family genes has not been performed in upland cotton. Currently, the *G. hirsutum* genome has been sequenced and provides the opportunity to perform a genome-wide analysis of the SnRK2 protein kinases family.

In the present study, we conducted a genome-wide survey of *G. hirsutum* molecular databases using SnRK2 protein sequences from *Arabidopsis*. We detected 20 putative *SnRK2* sequences in the *G. hirsutum* genome, named *GhSnRK2.1* to *GhSnRK2.20*. We analyzed their gene structures, chromosomal locations, expression profiles under exposure to abiotic stresses, and phylogenetic relationships. This is the first study to undertake a genome-wide analysis of *GhSnRK2s*. These results provide valuable information on *SnRK2* genes in *G. hirsutum* and will facilitate future studies of evolutionary relationships among cotton species.

## Results

### Identification of *SnRK2* genes in the *G. hirsutum* genome

A local BLASTP search was performed to identify SnRK2 members in the *G. hirsutum* genome using *Arabidopsis* SnRK2 protein sequences as the query sequence. The Hidden Markov Model profile of the Pkinase domain (PF00069.22) (http://pfam.sanger.ac.uk/) was used as a query to search the Pfam database using the program HMMER 3.0 with the default E-value. The serine/threonine protein kinase catalytic domains were determined using the online program SMART (http://smart.embl-heidelberg.de/) with the default E-value. We detected 20 predicted candidate SnRK2 family proteins that have complete serine/threonine protein kinase catalytic domains in the *G. hirsutum* proteome (Table 1). Eighteen *SnRK2* genes were mapped onto chromosomes but two *SnRK2* genes could not be conclusively localized to a chromosome. The genes were designated *GhSnRK2.1* to *GhSnRK2.20*. The length of the *SnRK2* coding sequence ranged from 999 to 1092 bp.

**Table 1:** Characteristics of SnRK2 in *Gossypium hirsutum*

Gene Name	Chromosomes	Start	End	Gene Length	Gene ID	Number of amino acids	Mol.mass (Da)	pI	CDS(bp)	Grand average of hydropathicity (GRAVY)
*GhSnRK2.1*	A01	447,830	450,835	3005	Gh_A01G0057	360	40,975.4	4.86	1083	−0.347
*GhSnRK2.2*	A02	16,299,920	16,301,838	1918	Gh_A02G0789	332	37,602.1	5.73	999	−0.194
*GhSnRK2.3*	A03	98,135,397	98,138,978	3581	Gh_A03G1684	363	41,269.7	4.80	1092	−0.347
*GhSnRK2.4*	A05	20,148,675	20,150,866	2191	Gh_A05G1922	339	38,495.8	5.53	1020	−0.382
*GhSnRK2.5*	A10	73,521,474	73,525,386	3912	Gh_A10G1380	357	40,937.4	5.79	1074	−0.492
*GhSnRK2.6*	A11	4,550,580	4,552,694	2114	Gh_A11G0474	341	38,756.1	5.07	1026	−0.325
*GhSnRK2.7*	A11	29,069,141	29,071,943	2802	Gh_A11G1757	361	40,906.5	4.98	1086	−0.257
*GhSnRK2.8*	A11	45,306,099	45,308,154	2055	Gh_A11G1858	341	38,616.1	5.57	1026	−0.362
*GhSnRK2.9*	scaffold2734_A11	24,966	27,913	2947	Gh_A11G3023	360	41,040.6	4.79	1083	−0.330
*GhSnRK2.10*	A12	18,241,166	18,244,267	3101	Gh_A12G0641	361	40,741.2	4.77	1086	−0.251
*GhSnRK2.11*	D01	395,483	398,406	2923	Gh_D01G0057	360	41,008.5	4.83	1083	−0.352
*GhSnRK2.12*	D02	14,597,151	14,599,078	1927	Gh_D02G0839	337	38,171.7	5.52	1014	−0.218
*GhSnRK2.13*	D02	65,195,858	65,199,438	3580	Gh_D02G2104	363	41,269.7	4.84	1092	−0.348
*GhSnRK2.14*	D05	20,185,702	20,187,904	2202	Gh_D05G2155	339	38,422.7	5.37	1020	−0.344
*GhSnRK2.15*	D10	17,134,725	17,138,661	3936	Gh_D10G1083	356	40,808.3	5.89	1071	−0.483
*GhSnRK2.16*	D11	4,160,991	4,163,929	2938	Gh_D11G0489	360	40,868.4	4.85	1083	−0.311
*GhSnRK2.17*	D11	4,806,770	4,808,880	2110	Gh_D11G0552	341	38,749.1	5.26	1026	−0.356
*GhSnRK2.18*	D11	32,580,911	32,582,968	2057	Gh_D11G2149	341	38,682.1	5.47	1026	−0.369
*GhSnRK2.19*	scaffold4551_D11	2394	5223	2829	Gh_D11G3472	361	40,892.5	4.98	1086	−0.257
*GhSnRK2.20*	D12	28,126,914	28,129,959	3045	Gh_D12G0859	361	40,798.3	4.82	1086	−0.269

The *SnRK2* genes differed substantially by the encoded protein size and its biophysical properties (Table 1). The SnRK2 protein sequences ranged from 332 to 363 amino acids in the case of *GhSnRK2.2* and *GhSnRK2.13*, respectively. The isoelectric point (pI) of all SnRK2 proteins was acidic, which indicates that SnRK2 proteins in cotton are rich in acidic amino acids. The GRAVY value of a protein is calculated as the sum of the hydropathy value of each residue, divided by the total number of residues present in the sequences. Positive and negative GRAVY scores reflect hydrophobicity and hydrophilicity, respectively [43]. The GRAVY scores of all *G. hirsutum* SnRK2 proteins were negative, which indicated that all were hydrophilic proteins, however, the degree of hydrophilicity exhibited a higher variability.

### Cis-element analysis in the promoter regions of *GhSnRK2* genes

To identify the putative cis-acting regulatory elements, 2000 bp of sequence upstream from the start codon was isolated. These regulatory elements included ABA-responsive elements (ABREs), low-temperature responsive elements (LTRs), defense and stress responsiveness elements (TC-rich repeats), salicylic acid responsive elements (TCA-elements), heat stress responsive elements (HSEs), MeJA-responsive elements (TGACG-motifs), MYB-binding sites (MBS), which are involved in drought-inducibility (Table 2).

**Table 2:** The cis-element analysis of *GhSnRK2* promoters

Gene	a	b	c	d	e	f	g
*GhSnRK2.1*	1	0	0	1	3	1	0
*GhSnRK2.2*	1	1	1	1	0	0	0
*GhSnRK2.3*	1	1	1	1	7	0	2
*GhSnRK2.4*	1	1	3	2	2	2	1
*GhSnRK2.5*	1	0	0	1	1	0	0
*GhSnRK2.6*	2	0	1	1	3	0	2
*GhSnRK2.7*	0	0	1	4	3	1	2
*GhSnRK2.8*	1	0	2	2	2	4	1
*GhSnRK2.9*	1	0	1	0	2	2	0
*GhSnRK2.10*	1	0	1	2	0	0	1
*GhSnRK2.11*	1	0	0	0	2	2	0
*GhSnRK2.12*	2	1	2	2	0	2	1
*GhSnRK2.13*	1	0	0	2	8	0	0
*GhSnRK2.14*	1	1	3	2	0	0	3
*GhSnRK2.15*	1	0	1	1	1	4	1
*GhSnRK2.16*	1	0	1	0	2	2	1
*GhSnRK2.17*	2	0	1	1	2	2	1
*GhSnRK2.18*	1	0	3	2	1	2	2
*GhSnRK2.19*	0	1	0	0	1	0	3
*GhSnRK2.20*	1	0	0	1	0	0	1

Numbers 1, 2, 3… represent the number of repeats of each cis-element whereas 0 indicates absence of the particular cis-element. Letters a, b, c… represent cis-elements. a: ABRE(CACGTG); b: LTR(CCGAAA); c: TC-rich repeats (ATTTTCTTCA); d: TCA-element(GAGAAGAATA); e: HSE(AAAAAATTTC); f: TGACG-motif(TGACG); g: MBS(CAACTG)

Plant hormone-responsive elements, including ABREs, TCA-elements and TGACG-motifs, were enriched in the upstream promoter regions of *GhSnRK2* genes. ABRE was the most abundant cis-acting hormone responsive element in the promoters of *GhSnRK2* genes. The genes, except *GhSnRK2.7* and *GhSnRK2.19*, contained ABRE-elements. TCA-elements and TGACG-motifs are involved in SA and MeJA responsiveness, respectively. In total, 16 members contained a TCA-element, whereas 11 members contained a TGACG-motif element. This indicates that *GhSnRK2* genes may respond to ABA, SA and JA.

The other important type of cis-acting elements in the upstream regions of *GhSnRK2* genes are environmental stress-related elements. In total, four types of elements were found that respond to four respective kinds of external environmental stresses. These were low-temperature responsive (LTR), stress-responsive TC-rich repeats, heat-stress-responsive HSEs and drought-responsive MBSs. Over half of the 20 *GhSnRK2* genes contained an HSE, MBS and TC-rich repeat (for defense and stress responses). Only six members (*GhSnRK2.2*, *GhSnRK2.3*, *GhSnRK2.4*, *GhSnRK2.12*, *GhSnRK2.14* and *GhSnRK2.19*) contained LTR-element. We hypothesized that external environmental stresses could induce the expression of *GhSnRK2* genes through their responsive cis-acting elements, further heightening the plants’ resistance to environmental stresses.

### Phylogenetic analysis and classification of the *GhSnRK2* gene family

**Fig. 1:**
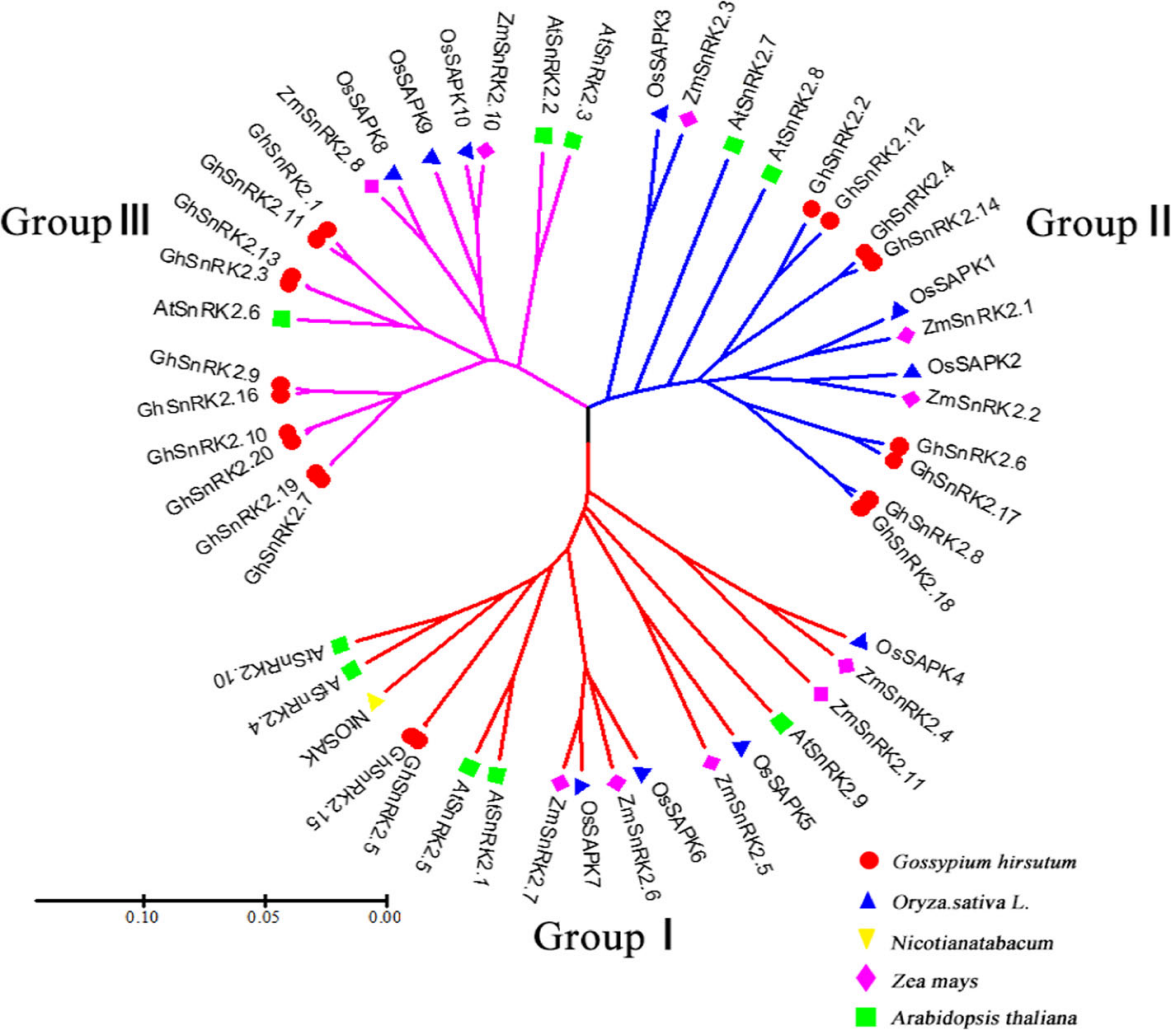
Phylogenetic analysis of SnRK2 proteins from Arabidopsis (SnRK), rice (SAPK), maize (ZmSnRK), tobacco (NtOSAK), and cotton (GhSnRK). Full-length amino acid sequences were aligned and phylogenetic tree was constructed by the UPGMA algorithm using MEGA5 software

We constructed a phylogenetic tree from a multiple alignment of SnRK2 protein sequences, comprising 20 GhSnRK2s from *G. hirsutum*, 10 SAPKs from rice, 10 SnRK2s from *Arabidopsis*, 11 ZmSnRK2s from maize, and 1 NtOSAK from tobacco. The SnRK2 proteins were clustered into three groups (Groups I, II and III; Fig. 1). Consistent with previous classification, all of the Arabidopsis SnRK2s were distributed among the three groups [27]. Our phylogenetic reconstruction showed that the GhSnRK2s were more closely grouped with SnRK2s from the dicots Arabidopsis and tobacco than with proteins from the monocots rice and maize. GhSnRK2.5 and GhSnRK2.15 belonged to Group I. Group II comprised of 8 members (GhSnRK2.2, GhSnRK2.4, GhSnRK2.6, GhSnRK2.8, GhSnRK2.12, GhSnRK2.14, GhSnRK2.17 and GhSnRK2.18). 10 members (GhSnRK2.1, GhSnRK2.3, GhSnRK2.7, GhSnRK2.9, GhSnRK2.10, GhSnRK2.11, GhSnRK2.13, GhSnRK2.16, GhSnRK2.19 and GhSnRK2.20) were included in Group III.

### Sequence alignment and C-terminal conserved motifs analysis

**Fig. 2:**
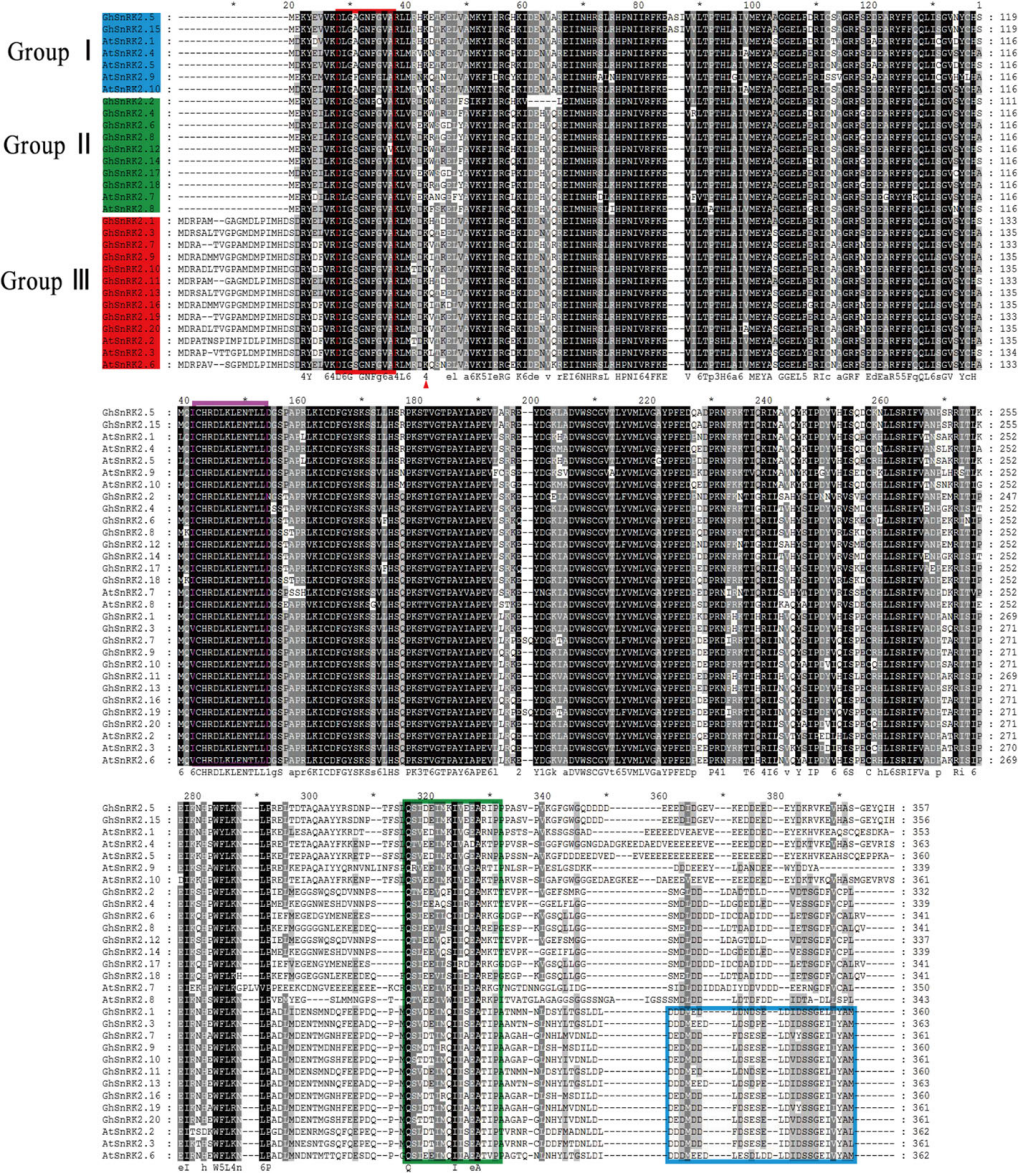
Multiple sequence alignment of the GhSnRK2 and AtSnRK2 proteins was obtained by DNAMAN software. Protein kinases ATP-binding region signature and serine/threonine protein kinases active-site signature are indicated by a *red box* and a *purple box*, respectively. DomainIand DomainIIat C terminus are marked by *green box* and *blue box*, respectively

We analyzed all the conserved motifs of GhSnRK2s and identified the pattern of amino acid residues conservation in their domains (Fig. 2). The N-terminal catalytic domain was highly conserved, containing an ATP-binding site and a protein kinase-activating site. The ATP-binding region contained I/LGXGXFGVA where a lysine residue serving as a binding site for ATP (red box). The serine/threonine protein kinase active-site signature V/ICHRDLKLENTLL contained an aspartic acid residue (the active site) (purple box). These two parts constituted the serine/threonine kinase domain. The regulatory C-terminal domain contained stretches of acidic amino acids. On the basis of these structural features, the SnRK2 kinases were divided into two classes, namely SnRK2a and SnRK2b, either poly-Asp (D) (SnRK2a) or poly-Glu (E) (SnRK2b).

The C-terminal domain consisted of two subdomains. Domain I was characteristic for all SnRK2 family members and is needed for activation by osmotic stress, i.e. the SnRK2-specific box (Gln-303 to Pro-318) (green box). Domain II was specific to the ABA-dependent SnRK2s and is needed for ABA responses, i.e. the ABA-specific box (Leu-333 to Met-362) (blue box) [44]. The ABA-specific box was present only in the members of Group III.

**Fig. 3:**
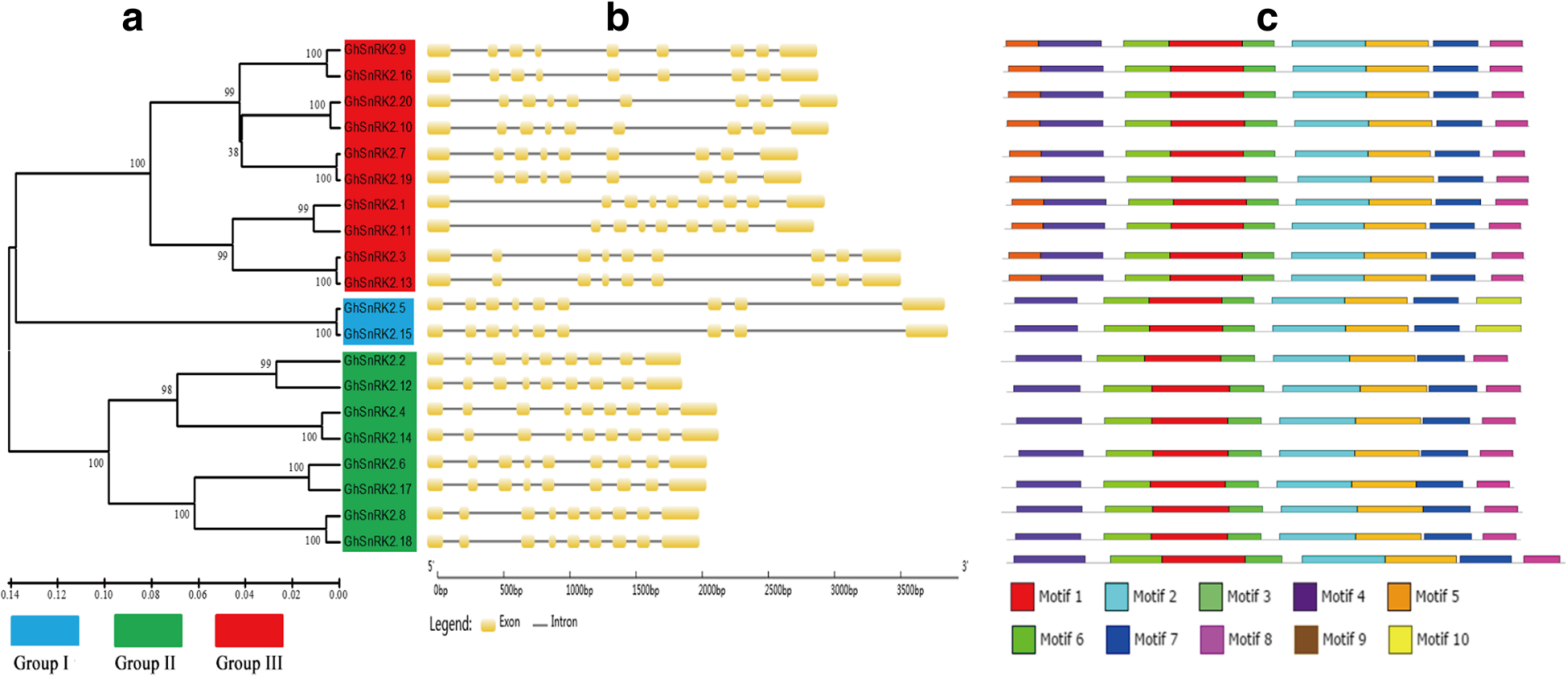
Phylogenetic relationship, exon-intron organizations, and motif analysis of GhSnRK2s. **a** Phylogenetic analysis of GhSnRK2s using by UPGMA method (**b**) Gene structure of the *GhSnRK2* family. Exons and introns are shown by filled boxes and single lines, respectively. **c** Analysis with MEME to investigate 10 conserved motifs of GhSnRK2 proteins. Each colored box represents conservation. Details are included with Supplementary

The GhSnRK2s showed 76.75% amino acid sequence identity, with highly conserved N-terminal kinase domains but divergent C-terminal domains. We employed MEME to detect conserved motifs in the GhSnRK2 family. Ten conserved motifs were scattered among each GhSnRK2 family (Fig. 3). All of the GhSnRK2 proteins shared the same seven motifs, designated motifs 1 to 7. Motif 4 contained the ATP-binding site. Motif 1 contained the serine/threonine protein kinase active-site. These two motifs constituted the N-terminal kinase domain. Motif 7 was located in the C-terminal Domain I, the SnRK2-specific box (Gln-303 to Pro-318). GroupIIISnRK2s, which are strongly activated by ABA, contain the ABA-specific box (Leu-333 to Met-362) involved in Motif 8. On the basis of previous reports, other conserved sequences in SNF1 were Arg(R)-Asp(D)-Leu(L) (residues 176 to 178 in the SNF1 protein), Asp(D)-Phe(F)-Gly(G) (residues 195 to 197) and Ala(A)-Pro(P)-Glu(E) (residues 219 to 221) [15], which were located in motif 1 and motif 6, respectively. Information for each motif, including site number and motif logo, is shown in (Additional file 1: Figure S1).

### Exon/intron organization of the cotton *GhSnRK2* gene family

Analysis of exons and introns arrangement can provide important insights into the evolution of gene families [45]. To investigate the exon/intron structure of the *GhSnRK2* genes, the coding and genomic sequences were compared, which showed that the genes contained nine exons interrupted by multiple introns (Fig. 3). In contrast to phylogenetic analysis, most members within the same group showed similar exon–intron structure and gene length. For example, in Group I, GhSnRK2.5 and GhSnRK2.15 showed 99.07% nucleotide sequence identity as indicated by the number of exons and similar intron phase patterns. This conservation of exon and intron numbers in each group strongly support the close evolutionary relationships of *GhSnRK2* genes. In addition, *GhSnRK2* genes in different groups usually varied in intron phase patterns and gene lengths.

### Chromosomal location and evolutionary history of the *GhSnRK2s* gene family

**Fig. 4:**
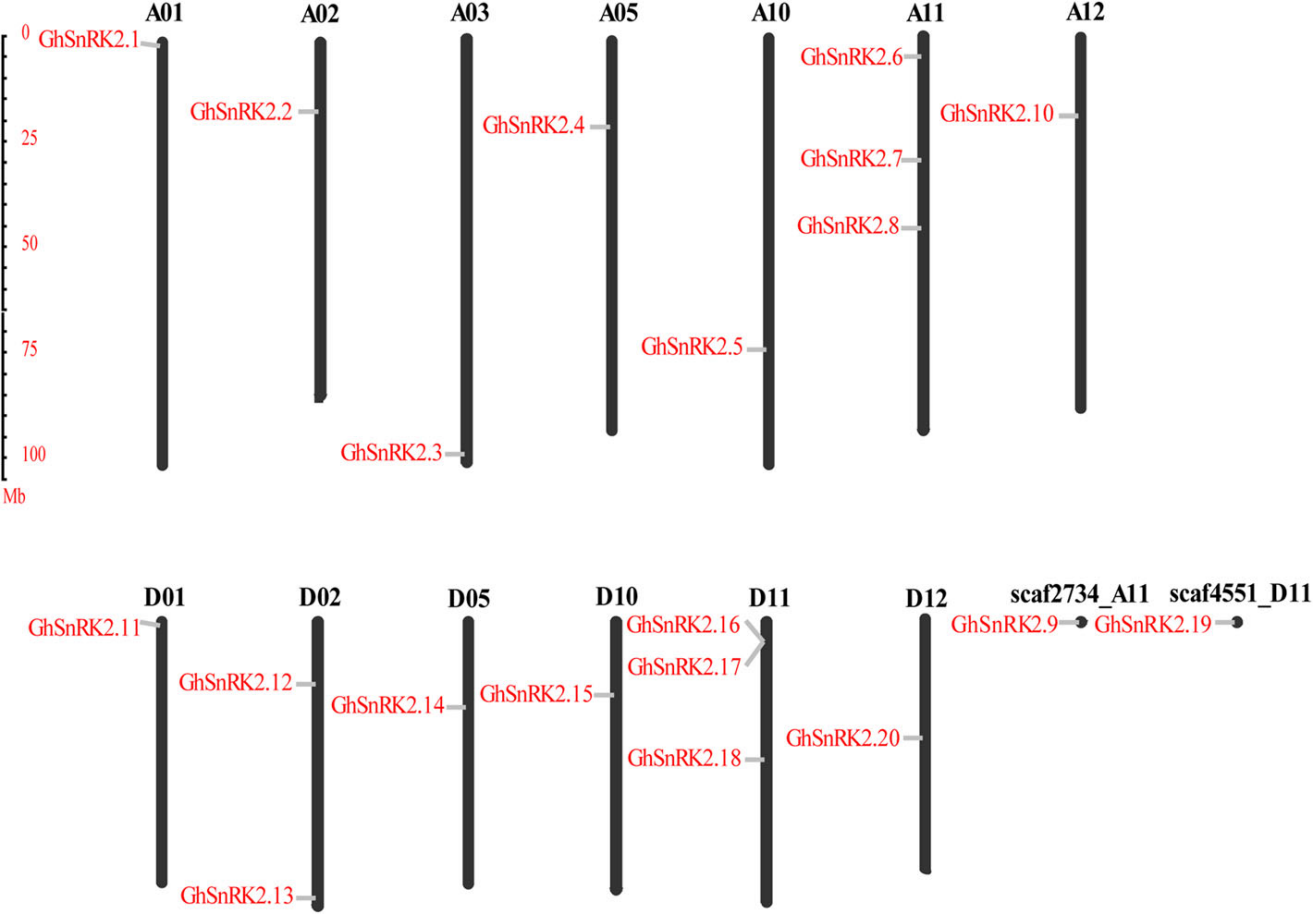
Chromosomal localization of *GhSnRK2* genes on *Gossypium hirsutum* chromosomes. The candidate *GhSnRK2* genes are designated *GhSnRK2.1* to *GhSnRK2.20* following their orders on chromosomes and from top to bottom

Among 20 cotton *SnRK2* genes, 18 genes were mapped onto 13 chromosomes and were named according to their order on the chromosomes (i.e., *GhSnRK2.1*, *GhSnRK2.2*, *GhSnRK2.3*, *GhSnRK2.4*, *GhSnRK2.5*, *GhSnRK2.6*, *GhSnRK2.7*, *GhSnRK2.8*, *GhSnRK2.10*, *GhSnRK2.11*, *GhSnRK2.12*, *GhSnRK2.13*, *GhSnRK2.14*, *GhSnRK2.15*, *GhSnRK2.16*, *GhSnRK2.17*, *GhSnRK2.18* and *GhSnRK2.20*) (Fig. 4). Two *GhSnRK2* genes that could not be conclusively mapped to any chromosome were named *GhSnRK2.9* and *GhSnRK2.19* (Fig. 4).

Nine *GhSnRK2* genes were located on A-subgenome chromosomes. Among these *GhSnRK2.1*, *GhSnRK2.2*, *GhSnRK2.3*, *GhSnRK2.4*, *GhSnRK2.5* and *GhSnRK2.10* were located on chromosome A01, A02, A03, A05, A10 and A12, respectively. *GhSnRK2.6*, *GhSnRK2.7* and *GhSnRK2.8* were all present on A11.Nine *GhSnRK2* genes were mapped to D-subgenome chromosomes. *GhSnRK2.11*, *GhSnRK2.14*, *GhSnRK2.15* and *GhSnRK2.20* were located on D01, D05, D10 and D12, respectively. D02 hosted two genes (*GhSnRK2.12* and *GhSnRK2.13*). Three genes were allocated to chromosome D11 (*GhSnRK2.16*, *GhSnRK2.17* and *GhSnRK2.18*). *GhSnRK2.9* and *GhSnRK2.19* were mapped to scaffold2734_A11 and scaffold4551_D11, respectively. This unbalanced distribution of *GhSnRK2* genes on chromosomes suggested that genetic variation existed in the evolutionary process.

In eukaryotes, presumably there might be a high similarity to among duplication mechanisms during the expansion of gene families; these mechanisms include tandem duplication, segmental duplication and whole-genome duplication [46]. To infer possible relationships between *GhSnRK2* genes and potential gene duplication within the *G. hirsutum* genome, we analyzed the occurrence of tandem duplication and segmental duplication during the evolution of this gene family. By calculating the similarity and the sequence coverage of the 20 *GhSnRK2* genes, we identified 10 pairs of genes (*GhSnRK2.1/GhSnRK2.11*, *GhSnRK2.2/GhSnRK2.12, GhSnRK2.3/GhSnRK2.13, GhSnRK2.4/GhSnRK2.14, GhSnRK2.5/GhSnRK2.15, GhSnRK2.6/GhSnRK2.17, GhSnRK2.7/GhSnRK2.19, GhSnRK2.8/GhSnRK2.18, GhSnRK2.9/GhSnRK2.16 and GhSnRK2.10/GhSnRK2.20*) that undergo segmental duplication (Table 3).

**Table 3:** The d_N_/d_S_ values for duplicate *GhSnRK2* genes

Paralogous	Amino acid sequence Identities (%)	d_S_	d_N_	d_N_/d_S_	Duplicate	Purifying selection
*GhSnRK2.1/2.11*	97.78	0.0812	0.0101	0.1247	segmental	Yes
*GhSnRK2.2/2.12*	93.18	0.0570	0.0261	0.4582	segmental	Yes
*GhSnRK2.3/2.13*	99.72	0.0234	0.0012	0.0522	segmental	Yes
*GhSnRK2.4/2.14*	98.53	0.0374	0.0066	0.1765	segmental	Yes
*GhSnRK2.5/2.15*	99.44	00240	0.0012	0.0521	segmental	Yes
*GhSnRK2.6/2.17*	97.36	0.0434	0.0113	0.2613	segmental	Yes
*GhSnRK2.7/2.19*	99.72	0.0258	0.0013	0.0492	segmental	Yes
*GhSnRK2.8/2.18*	98.83	0.0216	0.0052	0.2386	segmental	Yes
*GhSnRK2.9/2.16*	98.89	0.0074	0.0050	0.6781	segmental	Yes
*GhSnRK2.10/2.20*	99.17	0.0235	0.0037	0.1577	segmental	Yes

To assess the evolutionary history of the *GhSnRK2* gene family, synonymous (*d*_S_) and non-synonymous (*d*_N_) values were calculated using PAL2NAL. Nucleotide substitutions in protein-coding genes can be classified as synonymous or non-synonymous. Therefore, *d*_S_ and *d*_N_ values may allow ascertainment of whether selection has acted on protein-coding genes [47]. We estimated the *d*_N_/*d*_S_ values for the 10 pairs of segmental-duplicated genes (Table 3). The *d*_N_/*d*s ratios for the 10 pairs of genes were lower than 1, which suggested that purified selection acted on these duplicated gene pairs [48].

### Expression profiles of the *GhSnRK2* genes under abiotic stresses and ABA treatment

**Fig. 5:**
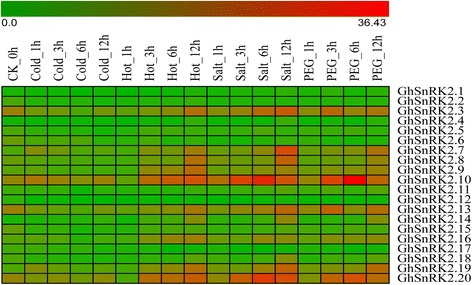
Expression analysis of *GhSnRK2* genes in *G.hirsutum* TM-1 under abiotic stresses. The RNA-Seq expression profiles of *G.hirsutum* TM-1 [51] were used to identify the relative expression levels of *GhSnRK2* genes. Levels of gene expression are depicted in different color on the scale. *Red* color represents high expression and *green* color represents low expression.The detail FPKM values are present in Additional file 2: Table S1 and Additional file 3: Table S2

*SnRK2* genes were shown to respond to abiotic stress [30, 32]. To understand expression patterns of these 20 *GhSnRK2* genes in *G.hirsutum*, we used publicly available transcriptome data to assess the expression under abiotic stresses conditions, i.e. cold, heat, salt and drought (Fig. 5). The analysis revealed that GhSnRK2 genes (*GhSnRK2.3/2.7/2.8/2.9/2.10/2.13/2.14/2.16/2.18/2.19/2.20*) responded to abiotic stresses, whereas the expression of other genes was not significantly altered under different stresses.

**Fig. 6:**
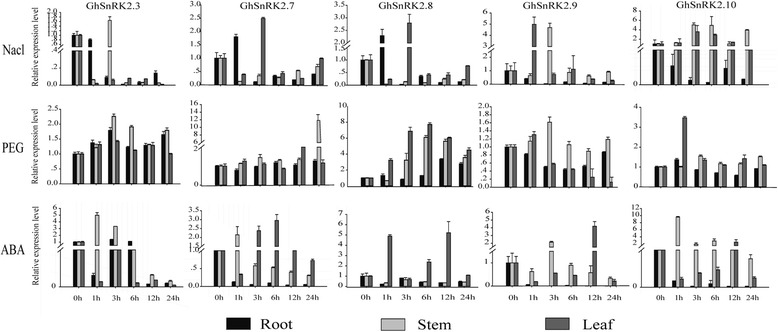
Expression analysis of five selected *GhSnRK2* genes using quantitative real-time RT-PCR (qRT-PCR) under Nacl, PEG, ABA treatment. The relative expression level of five selected *GhSnRK2* genes was normalized to the reference gene histone3 in different tissues. The transcripts in non-stressed was set as 1 for each gene in different tissues. The *bars* show the standard deviation of three technical repeats

Five *GhSnRK2* genes (*GhSnRK2.3/2.7/2.8/2.9/2.10*) were notably up-regulated under salt and PEG (Polyethylene Glycol**)** treatment based on the transcriptome data, were selected for further analysis by qRT-PCR. Furthermore we detected the expression patterns of five *GhSnRK2* selected genes in vegetative tissues (root, stem and leaf) (Fig. 6).

In response to NaCl treatment, the transcription of *GhSnRK2.3*, *GhSnRK2.7* and *GhSnRK2.8* peaked at 3 h. While based on the transcriptome data the expression of these three genes was maximum at 24 h under salt stress. The expression level of *GhSnRK2.10* in stem increased until 6 h, and then decreased at 12 h. This is consistent with transcriptome data that the expression of *GhSnRK2.10* peaked at 6 h after salinity treatment.

Under drought stress, the transcription of *GhSnRK2.3* in stem was increased until 3 h as well as the expression of *GhSnRK2.3* that peaked at 3 h under PEG treatment based on the transcriptome data. Transcript level of *GhSnRK2.10* in stems was slightly increased in response to dehydration. Meanwhile the expression level of *GhSnRK2.10* was maximum at 1 h in leaves. In contrast, *GhSnRK2.10* was notably up-regulated under PEG treatment in transcriptome data.

In response to exogenous ABA application, the transcript levels of *GhSnRK2.3* and *GhSnRK2.10* in stems were dramatically induced at 1 h. In leaves transcription of *GhSnRK2.10* decreased within the first 6 h, however transcript levels peaked at 12 h, and then declined at 24 h. Additionally, *GhSnRK2.8* expression was mainly induced in leaves and peaked at 12 h.

## Discussion

An understanding of the response of plants to water deficit is important in efforts to model cotton (*G.hirsutum*) growth [49]. Therefore it is necessary to identify novel genes that confer significantly enhanced high water-use efficiency. The SnRK2 protein family members are plant-specific serine/threonine kinases involved in response to abiotic stresses, especially drought and salinity [50]. Such protein kinases have been identified in many plant species. However, little information is available on this protein family in *G.hirsutum*. The release of the *G. hirsutum* genome sequence laid the foundation for comprehensive identification and characterization of the SnRK2 family in *G. hirsutum* [51]. Identification and analysis of *SnRK2* genes in *G. hirsutum* will prove to be a valuable tool for genetic improvement of allotetraploid *Gossypium* species.

### Identification of *GhSnRK2* genes

We identified 20 *SnRK2* genes in the *G. hirsutum* genome, where 10 genes belonged to the A subgenome and the others to the D subgenome. Compared with other comprehensively surveyed plant SnRK2s (10 SnRK2 family genes have been identified in Arabidopsis, 10 in rice, 11 in maize, four in soybean, six in grape, three in tobacco, 10 in sorghum and 12 in apple), the *GhSnRK2* family is the largest with 20 phylogenetically expanded genes. The striking expansion and diversification of the *GhSnRK2* family suggests that these SnRK2s play crucial roles in the environmental adaptability and physiological maintenance in *G. hirsutum*, which can survive drought stress and other stressful environments.

### Evolution of the *SnRK2* family genes

Phylogenetic analysis and comparative genomic analyses are usually conducted to gain insight into evolutionary relationships of genes or species. To further evaluate the evolutionary relationships of *GhSnRK2s* with related genes from other plant species, we constructed an unrooted phylogenetic tree from an alignment of the full-length protein sequences. The phylogenetic analysis divided the *GhSnRK2* family members into three different groups. The orthologous genes from rice, maize, Arabidopsis, tobacco and cotton were clustered in the same group, implying that SnRK2s originated before the divergence of monocots and dicots. Evolutionary studies based on SnRK2 protein sequences from green algae (*Chlamydomonas reinhardtii*), moss (*Physcomitrella patens*), fern (*Selaginella moellendorffii*), and angiosperms (including Arabidopsis and rice) showed that higher plants possess all three SnRK2 groups. Moss only possesses SnRK2s belonging to Group III and fern kinases belong to Group II. Green algal SnRK2s are distinct from those of land plants [52–55]. These results indicate a possible evolutionary pattern of plant SnRK2s: Group III is an ancient form of land plant SnRK2, whereas Group I arising before angiosperms of most recent origin. The size of Group I SnRK2s in cotton is smaller when compared with that of Arabidopsis, rice and maize, which indicates that the number of novel Group I proteins may have increased in these herbaceous plants during evolution.

From the perspective of the primary structure of the protein, phylogenetic analysis of the amino acid sequence provides information on evolutionary relationships. Previous reports have indicated that most SnRK2s from higher plants include nine exons [33]. We found that our GhSnRK2s also have nine exons while intron phase patterns and gene lengths were contrary to other species. This finding may indicate that in dicot and monocot plant lineages the SnRK2 members have experienced uneven deletion of introns leading to the variation in intron size and number. In addition, on the basis of the chromosomal arrangement of genes and comparative analysis of gene location, length, and structure, we identified 10 paired segmental-duplicated *SnRK2* genes in the *G. hirsutum* genome. Preliminary analyses revealed that gene duplication and subsequent divergence are the main contributors to evolutionary momentum [56]. Usually, the criteria for inferring a gene duplication event are (1) the length of the alignment sequence covers ≥80% of the longest gene, and (2) the similarity of the aligned regions is ≥70% [57]. Two or more genes located on the same chromosome result from tandem duplication, whereas gene duplication between different chromosomes is designated as a segmental duplication event [58]. Two pairs of *SnRK2* genes in Arabidopsis (*AtSnRK2.1/AtSnRK2.5*, and *AtSnRK2.2/AtSnRK2.3*) were identified as segmental duplication pairs. In rice, *SAPK1/SAPK2* and *SAPK4/SAPK5* have undergone segmental duplication [31]. The current study suggests that *G. hirsutum* is similar to Arabidopsis and rice in having experienced segmental duplication events. Gene duplication plays a key role facilitating the evolution of organisms by creating the raw genetic material that is modified by subsequent natural selection pressure [59]. These segmental duplications may have contributed greatly to the expansion and evolution of the *SnRK2* gene family in *G. hirsutum*. It is speculated that the existence of such genes in the *G. hirsutum* genome might have been conducive to the structural and functional innovations by which cotton has been able to adapt to adverse environments.

### Regulatory elements in the promoters of *GhSnRK2* genes

Gene expression pattern can provide important clues to gene functions, which are believed to be associated with divergence in the promoter region [60]. Cis-acting regulatory elements contained in gene’ promoter regions play key roles in conferring the developmental and environmental regulation of gene expression. Based on previous reports, Group III genes were strongly activated by ABA, and in our group classification, 10 genes (*GhSnRK2.1*/*2.3*/*2.7*/*2.9*/*2.10*/*2.11*/*2.13*/*2.16*/*2.19*/*2.20*) belonged to Group III. ABA-responsive elements were found all of the members of Group III, expect *GhSnRK2.7* and *GhSnRK2.19*. ABA-responsive elements were also found in the promoters of GhSnRK2s in other groups. For example, *GhSnRK2.5* and *GhSnRK2.15*, which contain ABRE elements, belong to Group I.

Environmental stress-related elements were abundant in the *GhSnRK2s*. The regulation of adapting to certain external environmental conditions, such as drought, heat and low temperature, through *SnRK2*s has been well studied [23, 30, 37]. For *GhSnRK2*s, most members contained LTR, HSE and MBS elements. Moreover, many cis-elements were located at adjacent sites. There are two MBS elements in the promoter of *GhSnRK2.3*, which may enhance its drought responsiveness.

### C-terminal motifs might contribute to the ABA response

The amino acid sequences of all SnRK2s contain highly conserved N-terminal kinase domain and a regulatory C-terminal domain with an ‘acidic patch’ region. It is further divided into two subgroups based on the composition of the C-terminal domain: SnRK2a (rich in glutamic acid) and SnRK2b (rich in aspartic acid) [20]. In *Arabidopsis*, the acidic patch is aspartate-rich in SnRK2 members of Groups II and III but glutamate-rich in Group I members [53, 61]. Only aspartate-rich SnRK2s can be activated by ABA. In our protein motif analysis, an acidic patch region was also present in the C-terminal motifs of GhSnRK2s. Motif 10 (specific to Group I) is poly-glutamate and GhSnRK2.5/GhSnRK2.15 may not be activated by ABA. On the basis of previous reports, Group I comprised kinases were not activated by ABA, Group II kinases were not activated or activated very weakly by ABA (depending on plant species), and Group III proteins were strongly activated by ABA [28, 62–64]. In Group III, C-terminal Domain II is poly-aspartate involved in motif 8. Moreover, members of Group III in cotton (GhSnRK2.1/2.3/2.7/2.9/2.10/2.11/2.13/2.16/2.19/2.20) contain an ABA-specific box (Leu-333 to Met-362) and may be strongly activated by ABA. Although members of Group II lacked an ABA-specific box, they were rich in aspartate, thus Group II SnRK2s may be weakly activated by ABA.

### Some *GhSnRK2* genes respond to abiotic stresses and ABA treatment

Numerous studies demonstrated that SnRK2s are involved in multiple abiotic stress responses. In the present study we determined the dynamic transcriptional changes of *GhSnRK2* genes under drought and salinity stresses and ABA treatment. Drought resistance of Arabidopsis plants is influenced by *AtSnRK2.8*, which belongs Group II [30]. As determined by our real-time PCR analysis, *GhSnRK2.8*, which belongs to Group II, was induced in leaves at 3 h and 6 h after PEG treatment. In Arabidopsis, members of Groups II and III are reported to be involved in ABA-dependent signaling pathways [27, 65]. *GhSnRK2.10*, a member of Group III, also responded quickly to ABA treatment at 1, 3 and 6 h in stems. In rice, many *SAPK* genes are differentially regulated by mannitol, NaCl and ABA in different organs [62]. Salinity and drought induced elevated expression levels of *SAPK3*, *SAPK8* and *SAPK9* in rice [32]. We noted that *GhSnRK2.3* showed 82.31% amino acid identity with *SAPK8*. Thus, we speculate that *GhSnRK2.3* involved in regulation of salt stress tolerance in cotton. In many plant species, such as *Arabidopsis*, rice and maize, it is evident that SnRK2 proteins function as transcriptional activators in ABA-signaling mechanisms in response to abiotic stresses such as drought and salinity [26]. The expression of *GhSnRK2* genes is induced by drought, salinity and ABA treatments, which may be indicative of potential roles in stress responses.

## Conclusions

Although the function of certain *SnRK2* genes of *Arabidopsis* and rice has been clearly demonstrated, the functions of *SnRK2* genes in *G. hirsutum* are still elusive. In the present research, we performed a genome-wide analysis of the *SnRK2* gene family in *G. hirsutum*, including examination of gene structure, evolutionary relationships, and transcriptional changes in response to stress treatments. *GhSnRK2* genes were identified, which are distributed in different evolutionary lineages among higher plants. The expansion of the SnRK2 family in *G. hirsutum* might be as result of segmental duplication. Our expression analysis revealed differential responses among the *GhSnRK2* genes to abiotic stresses. The present genomic and bioinformatics analyses of *GhSnRK2* genes provide a solid foundation for further investigation of *GhSnRK2* gene functions. In addition, these results may help in genetic improvement of cotton stress tolerance.

## Methods

### Identification of *SnRK2* genes in *Gossypium hirsutum*

We downloaded the *G. hirsutum* genome sequence and the proteome sequences from the CottonGen database (http://www.cottongen.org/specises/Gossypium_hirsutum/nbi-AD1_genome_v1.1, 51]. A local BLASTP algorithm-based search with the blast-2.2.9 program, which was downloaded from the National Center for Biotechnology Information (NCBI) (ftp://ftp.ncbi.nlm.nih.gov/blast/executables/blast+/2.2.19/), was performed to identify complete SnRK2 members, using *Arabidopsis* SnRK2 sequences as query. The E- value threshold for BLASTP was set at 1e^−10^ to obtain the final dataset of SnRK2 proteins. Redundant sequences were removed. Then, the Pfam (http://pfam.sanger.ac.uk/search) and SMART (http://smart.embl-heidelberg.de/) databases were used to confirm each predicted GhSnRK2 protein sequence [66, 67]. The sequences of 10 rice and 10 *Arabidopsis* SnRK2 proteins were obtained from the Rice Genome Annotation Project (http://rice.plantbiology.msu.edu/) and the Arabidopsis Information Resource (TAIR; http://www.arabidopsis.org/) databases, respectively. The biophysical properties of the encoded proteins were computed using the ExPASy ProtParam tool (http://us.expasy.org/tools/protparam.html).

### Gene structure and C-terminal conserved motifs analysis

Structural information for the *GhSnRK2* genes, including chromosomal location and gene length, were obtained from the CottonGen databases. Exons and introns were predicted by comparing the coding sequences with genomic sequences using the gene structure display server (GSDS program 2.0) (http://gsds.cbi.pku.edu.cn/) [68]. Conserved motif prediction was performed using the MEME (http://meme-suite.org/) program. The analysis was performed with the following parameters: number of unique motifs: 10; and maximum and minimum search widths: 50 and 16, respectively.

### Mapping *SnRK2* genes on cotton chromosomes, and estimating synonymous (*d*_S_) and non-synonymous (*d*_N_) substitution rates

The chromosomal distributions of *GhSnRK2* genes were obtained based on genome annotation. The Mapchart 2.2 software was used to visualize the distribution of the *GhSnRK2* genes on the 13 *G. hirsutum* chromosomes.

The d_S_ and d_N_substitution rates were calculated with the PAL2NAL web server (http://www.bork.embl.de/pal2nal#RunP2N), which uses the CODEMAL program of PAML [69].

### Sequence alignment and phylogenetic tree construction

An alignment of multiple SnRK2 protein sequences from *Arabidopsis thaliana*, *Zea mays*, *Oryza sativa* and *Nicotiana tabacum* was generated using the ClustalW program [27, 31, 33, 62]. A neighbor-joining analysis of the generated alignment was performed using the unweighted pair-group method with arithmetic mean algorithm to construct an unrooted phylogenetic tree. Support for the tree topology was assessed by performing a bootstrap analysis with 1000 replicates. The tree was visualized with MEGA 5.05 software [70].

### Retrieval and analysis of promoter sequences

The *G. hirsutum* genome sequences were used to retrieve the promoter sequences (2 kb upstream of the start codon) of the *GhSnRK2* genes. The analysis of the *GhSnRK2* promoters was carried out using the PlantCARE database (http://bioinformatics.psb.ugent.be/webtools/plantcare/html/) [71].

### Gene expression analysis

The expression levels of *GhSnRK2* genes were measured using the RNA-sequencing data of *G.hirsutum* TM-1 acquired under four different stresses (cold, heat, salt and drought), which were downloaded from the NCBI Gene Expression repository under accession number PRJNA248163 (Additional files 2 and 3) (http://www.ncbi.nlm.nih.gov/bioproject/PRJNA248163/).

### RNA isolation and the qRT-PCR analysis

To examine expression profiles of *GhSnRK2* genes in different tissues and in response to abiotic stresses, upland cotton with three or four leaves were submerged in 10% PEG6000, 150 mM NaCl, or 100 μM ABA solutions. Samples were collected from the root, stems and leaves at 0, 1, 3, 6, 12, and 24 h after treatment. Samples collected at 0 h were used as controls. All samples were frozen immediately in liquid nitrogen and kept at −80 °C for total RNA extraction. Total RNA was extracted from the samples using the RNAprep Pure Kit (For Plants) (TIANGEN, Beijing, China). To remove genomic DNA contamination, the RNA samples were treated with DNase I. Gel electrophoresis and a Nanodrop2000 nucleic acid analyzer were employed to detect the quality of the RNA. First-strand cDNA was synthesized based on reverse transcription of 1 μg RNA digested by DNase I using the PrimeScript™ RT Reagent Kit (Takara, Dalian, China). PCR amplifications were performed using SYBR® Premix Ex Taq™ (Takara). For real-time PCR, gene-specific primers were designed using Primer 5.0 (Additional file 4). The cotton *histone3* gene (GenBank accession no. AF024716) was used as the internal reference gene [72].

For the qRT-PCR assay, cDNA was diluted to 100 ng/μl with ddH_2_O. The qRT-PCR reaction was carried out on an ABI 7900 HT Real-time PCR System (Applied Biosystems). The reaction system (in a total volume of 20 μl) contained 10 μl SYBR® Premix Ex Taq™ (2×), 0.4 μl of each primer (10 μM), 0.4 μl ROX Reference Dye (50×), 1 μl template (about 100 ng/μl), and ddH_2_O to make up the total volume. The protocol was performed as follows: pre-denaturation at 95 °C for 30 s (step 1), denaturation at 95 °C for 5 s (step 2), primer annealing/extension and gathering of the fluorescent signal at 60 °C for 1 min (step 3), followed by 40 cycles of step 2. Three biological replicates, each consisting of three technical replicates, were analyzed. The relative expression levels of the *SnRK2* genes were calculated using the 2^−△△Ct^ method [73].

## Additional files

Additional file 1: Figure S1. Details of conserved motifs detected among members of the GhSnRK2 protein family by MEME. (TIFF 1466 kb)

Additional file 2: Table S1. FPKM values under abiotic stresses. (XLSX 17 kb)

Additional file 3: Table S2. Gene accession number and samples information of transcriptome data used in our research. (XLSX 10 kb)

Additional file 4: Table S3. List of the primers used for quantitative real-time PCR in this study. (XLSX 10 kb)

## Abbreviations

*GhSnRK2s*: Genes in *Gossypium hirsutum* L.(AADD)
SnRK2: Sucrose non-fermenting-1-related protein kinase 2
