# Supplementary figures and images for: Genome-wide identification and characterization of SnRK2 gene family in cotton (Gossypium hirsutum L.)
Source: BMC Genet. 2017 Jun 12;18:54. doi: 10.1186/s12863-017-0517-3 (PMC5469022; doi:10.1186/s12863-017-0517-3)

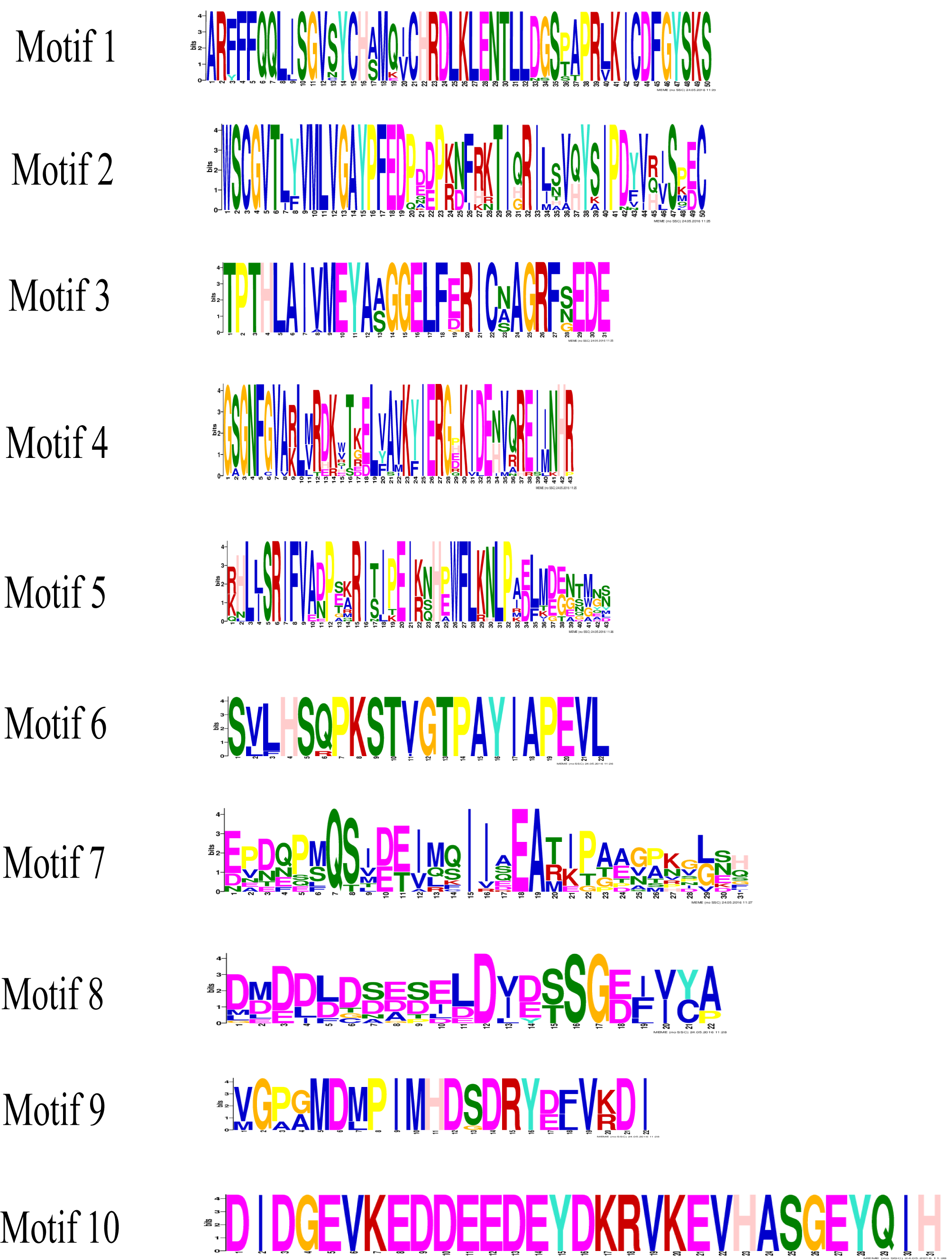

Supplement: Supplementary file 1 — Details of conserved motifs detected among members of the GhSnRK2 protein family by MEME. (TIFF 1466 kb) [file 12863_2017_517_MOESM1_ESM.tif]
